# Incidental Finding of Ascaris in Peritoneal Cavity During Laparotomy for Blunt Trauma Abdomen: A Rare Case

**DOI:** 10.7759/cureus.24970

**Published:** 2022-05-13

**Authors:** Savijot Singh, Prem Chand, Shivanshu Kundal, Goldendeep Singh, Deepam Kundal

**Affiliations:** 1 General Surgery, Maharishi Markandeshwar Medical College and Hospital, Solan, IND; 2 General Surgery, Government Medical College & Rajindra Hospital, Patiala, IND; 3 Psychiatry, Maharishi Markandeshwar Medical College and Hospital, Solan, IND

**Keywords:** ascaris, laparotomy, perforation, blunt trauma, intraperitoneal ascariasis, asymptomatic

## Abstract

*Ascaris* migration from the intestine into the peritoneal cavity is rarely seen and the usual presentation is the acute abdomen. Our case report is of a young male who got admitted after a roadside accident with polytrauma including blunt trauma abdomen. When the patient was taken up for exploratory laparotomy, a freely lying tubular structure was noticed in the pelvis and small intestinal perforation. On inspection, it turned out to be an *Ascaris* worm. This is a case report of a rare presentation of *Ascaris lumbricoides *with jejunal perforation following blunt trauma. This blunt trauma could have been the cause of an intestinal perforation resulting from a concealed presence of an impending *Ascaris *perforation.

## Introduction

*Ascaris lumbricoides* is one of the most common helminthic infections throughout the world with the highest prevalence in developing countries like India, Bangladesh, and China. Although rare, perforation peritonitis is a known complication of ascariasis [1]. It is postulated that the *Ascaris* produces a lytic secretion, and this together with the nibbling effect of the head of the *Ascaris* can lead to perforation of the normal bowel wall [1].

This is a report of a rare case of *Ascaris lumbricoides*-associated intestinal perforation with blunt abdominal trauma. Though the clinical presentation is usually delayed if there is mild trauma, this mild trauma could have led to intestinal perforation, accelerating the already present impending ascariasis perforation [1].

## Case presentation

**Figure 1:**
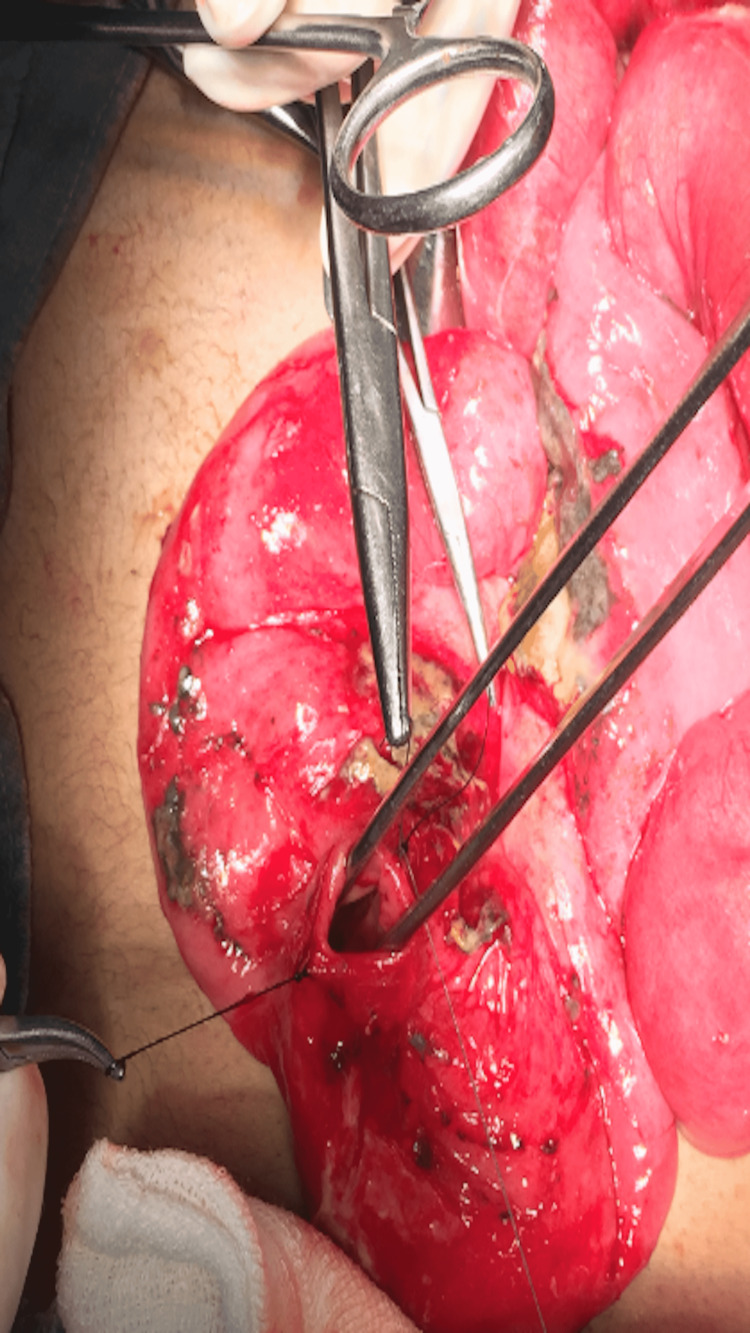
Perforation site in small intestine

**Figure 2:**
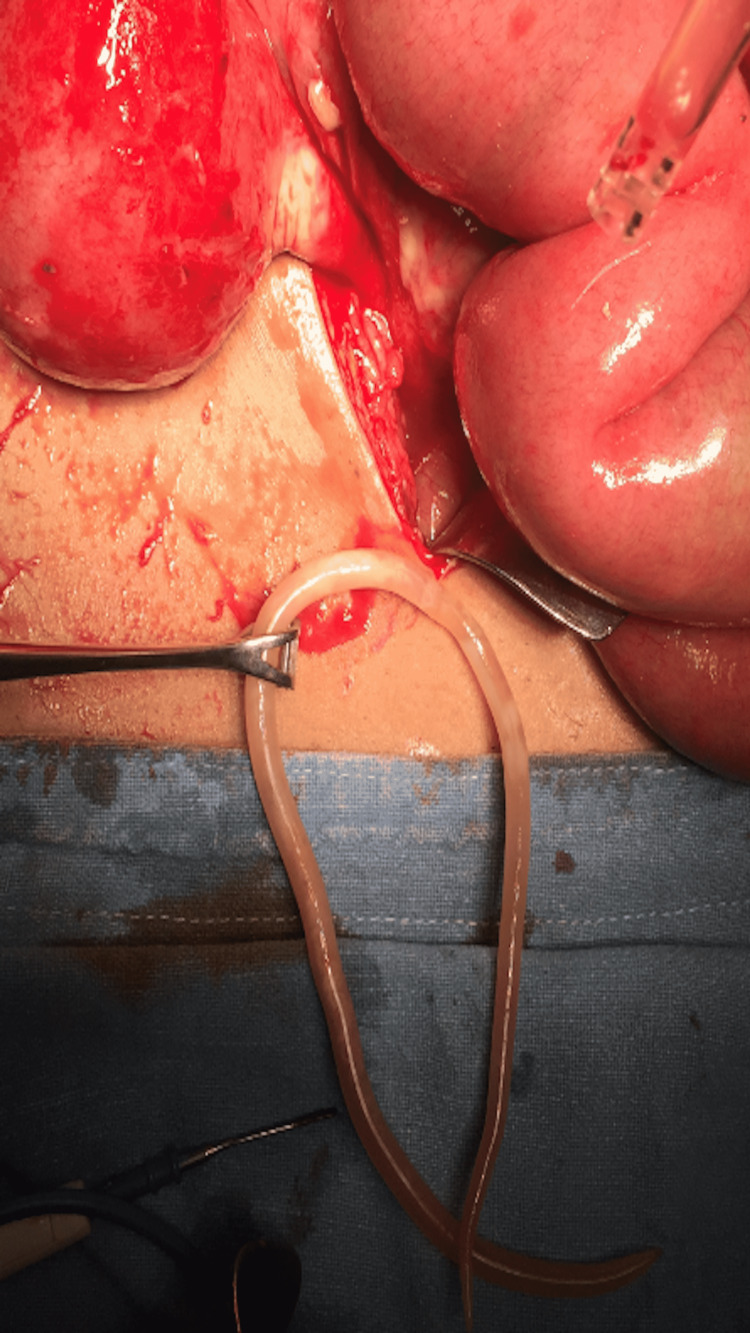
Ascaris worm found in pelvis

A 28-year-old male presented to the casualty after a roadside accident with polytrauma. On examination, the patient had multiple fractures of the femur, tibia, radius, and facial injuries. A history of blunt trauma to the abdomen was present. No complaint of pain in the abdomen on presentation. The abdomen was soft and non-tender. Ultrasonography and x-ray of the abdomen were grossly normal. On the third day of admission, the patient complained of bleeding per rectum along with pain abdomen. On examination, the abdomen was distended but no tenderness or mass was palpable. Contrast-enhanced CT (CECT) abdomen showed pneumoperitoneum, small bowel distended with multiple air-fluid levels, the largest being 5.1 cm, smooth narrowing of distal ileum stricture with moderate ascites. Urgent explorative laparotomy was planned, which was done under general anaesthesia with endotracheal intubation. During laparotomy, a single 1x1 cm perforation was found near the distal end of the jejunum on the anti-mesenteric border ([Figure 1). The collection was noticed in the pelvis and left paracolic gutter. *Ascaris* was found in pelvic collection roughly 20cm in length (Figure 2). *Ascaris* was extracted out and perforation was repaired in two layers with Vicryl 3-0 and silk 3-0 round body (RB) after a thorough peritoneal wash with saline. Two drains were placed in the pelvis and Morison's pouch. Postoperatively the patient was given a triple antibiotic cover and recovered well. The patient was allowed oral intake of food on postoperative day three. On review of the patient’s history, it was noticed that he frequently used to eat from street vendors. The history ruled out worms in stool, abdominal pain, or any history of intestinal perforation. He was treated with a course of albendazole.

## Discussion

*Ascaris lumbricoides* is one of the most common helminthic infections worldwide with maximum prevalence in developing countries [1]. Ascariasis has varied clinical presentations but generally runs a benign course. The intestinal perforation by normal worms is of two types, primary and secondary. In the primary perforation, the *Ascaris* perforates the healthy intestine, while in the secondary type there is an association of intestinal disease like enteric fever or weakness in the intestinal wall. Although rare, perforation peritonitis is a known complication of ascariasis. In most of these cases, co-existing bowel pathologies like typhoid, amoebiasis, Meckel's diverticulitis, ischemic necrosis due to volvulus, and trauma play a synergistic role in causing the perforation [2,3]. It has been postulated that the *Ascaris* produces a lytic secretion that helps in the nibbling effect of the *Ascaris* head and ischemia from pressure by the mass of worms in the small intestine leads to perforation of the normal bowel wall [1,2].

Injury to the intra-abdominal structures is primarily by either compression forces or deceleration forces. The compressive forces lead to deforming of hollow organs and temporarily increase intraluminal pressure, resulting in a perforation. Stretching and linear shearing between relatively fixed and free objects is caused by decelerating forces [4]. In our case, it is suggested that trauma might have precipitated an impending perforation due to ascariasis [5].

Albendazole 400 mg single dose orally is the drug of choice. Paralyzing vermifuges (eg, pyrantel pamoate, piperazine, ivermectin) are avoided in patients with intestinal obstruction because paralyzing the worms may lead to difficulty during surgery. In such cases, albendazole and mebendazole are preferred [1].

## Conclusions

In this case, blunt trauma along with an impending perforation led to jejunal perforation and symptoms associated with it. Perforation was repaired primarily. Therefore, this rare case has highlighted the chances of blunt trauma leading to perforation of the small intestine of an impending perforation by *Ascaris* as seen in this patient.
